# Generation of a conditional cellular senescence model using proximal tubule cells and fibroblasts from human kidneys

**DOI:** 10.1038/s41420-024-02131-y

**Published:** 2024-08-14

**Authors:** Xiaohang Shao, Huaming Xu, Hyojin Kim, Sadaf ljaz, Fabian Beier, Vera Jankowski, Michaela Lellig, Lucia Vankann, Jan Niklas Werner, Lu Chen, Susanne Ziegler, Christoph Kuppe, Martin Zenke, Rebekka K. Schneider, Sikander Hayat, Turgay Saritas, Rafael Kramann

**Affiliations:** 1 Department of Nephrology and Clinical Immunology, RWTH Aachen University, Medical Faculty, Aachen, Germany; 2 Department of Cell Biology, Institute of Biomedical Engineering, RWTH Aachen University Medical School, Aachen, Germany; 3 Helmholtz Institute for Biomedical Engineering, RWTH Aachen University, Aachen, Germany; 4 Department of Hematology, Oncology, Hemostaseology, and Stem Cell Transplantation, Faculty of Medicine, RWTH Aachen University, Aachen, Germany; 5 Center for Integrated Oncology Aachen Bonn Cologne Düsseldorf (CIO ABCD), Aachen, Germany; 6 Institute for Molecular Cardiovascular Research (IMCAR), RWTH Aachen University, Aachen, Germany; 7 Institute of Cell and Tumorbiology, RWTH Aachen University, Medical Faculty, Aachen, Germany; 8 Oncode Institute, Erasmus Medical Center, Rotterdam, The Netherlands; 9 Department of Internal Medicine, Nephrology, and Transplantation, Erasmus Medical Center, Rotterdam, The Netherlands

**Keywords:** End-stage renal disease, Senescence

## Abstract

Emerging evidence highlights cellular senescence’s pivotal role in chronic kidney disease (CKD). Proximal tubule epithelial cells (PTECs) and fibroblasts are major players in CKD and serve as cellular sources of senescence. The generation of a conditionally immortalized human kidney cell model would allow to better understand the specific mechanisms and factors associated with cellular senescence in a controlled setting, devoid of potential confounding factors such as age and comorbidities. In addition, the availability of human kidney cell lines for preclinical research is sparse and most cell lines do not reflect their in vivo counterparts due to their altered behavior as immortalized cancer-like cells. In this study, PTECs and fibroblasts from human kidneys were isolated and transduced with doxycycline-inducible simian virus 40 large T antigen (SV40LT) vector. By comparing their gene expression with single-cell RNA sequencing data from human kidneys, the newly produced human kidney cell lines demonstrated significant resemblances to their in vivo counterparts. As predicted, PTECs showed functional activity and fibroblasts responded to injury with fibrosis. Withdrawal of the immortalizing factor doxycycline led to p21^+^ cell-cycle arrest and the key hallmarks of senescence. The obtained senescence gene set largely overlapped between both cell lines and with the previously published SenMayo set of senescence-associated genes. Furthermore, crosstalk experiments showed that senescent PTECs can cause a profibrotic response in fibroblasts by paracrine actions. In 76 human kidney sections, the number of p21^+^ cells correlated with the degree of fibrosis, age and reduced glomerular filtration, validating the role of senescence in CKD. In conclusion, we provide a novel cellular ex vivo model to study kidney senescence which can serve as a platform for large scale compounds testing.

## Introduction

Chronic kidney disease (CKD) is a growing public health problem affecting more than 10% of people worldwide [1]. Accumulating evidence indicates that cellular senescence, an irreversible situation involving cell-cycle arrest, contributes to CKD [2]. In particular, proximal tubule epithelial cells (PTECs) are the major location of senescent cells after kidney injury or during aging [3]. Senescent cells obtain a senescence-associated secretory phenotype (SASP) and secrete numerous proinflammatory and profibrotic cytokines that can adversely affect neighboring healthy cells [4]. It is likely that the SASP in injured PTECs is a key mechanism through which fibroblast activation drives kidney fibrosis in CKD [5–8]. As interstitial fibrosis correlates with kidney function and preclinical data suggest that targeting fibrosis can stabilize kidney function, fibrosis is widely accepted as a therapeutic target for CKD [9]. Therefore, investigating the role of PTECs and fibroblasts in cellular senescence and the crosstalk between these two cell types will be highly important for understanding SASP-mediated kidney fibrosis. Moreover, senescent cells in the kidney may represent novel targets for preventing the progression of CKD [10]. However, robust human kidney in vitro cell models for senolytic drug screening are lacking, as most cell lines do not recapitulate their in vivo counterparts due to their altered behavior as immortalized cancer-like cells.

In the present study, we aimed to generate a conditionally immortalized cell model by transducing a doxycycline-inducible simian virus 40 large T antigen (SV40LT) vector into human PTECs and kidney fibroblasts. SV40LT is commonly introduced into primary cells to establish an immortalized cell line [11]. The T antigen binds and inhibits several proteins, including the tumor suppressor p53 and the retinoblastoma suppressor gene (pRB), to promote cell division [12]. Creating a conditional model enables the proliferation of cells under the influence of doxycycline-induced T antigen expression, maintaining a state of conditional immortality. Additionally, this approach provides the opportunity to investigate cells that will enter the senescence process upon the removal of doxycycline, as these cells exhibit characteristics similar to those of primary cells. This cell model would also enable the investigation of cellular senescence within a controlled environment, eliminating potential confounding factors such as age and comorbidities. Here, we demonstrate that the newly generated human kidney cell lines indeed exhibit significant similarities to their in vivo counterparts, as evidenced by comparisons with publicly available human kidney single-cell data from the Kidney Precision Medicine Project (KPMP) [13] and by the use of functional assays. Furthermore, the withdrawal of doxycycline led to the expression of a senescence signature similar to the SenMayo consensus signature [14] in both PTECs and fibroblasts. We were able to model the cellular crosstalk between senescent PTECs and fibroblasts, indicating that senescent PTECs can cause a profibrotic response in fibroblasts through paracrine actions. Thus, we present a newly developed scalable platform for investigating kidney senescence. This platform holds promise for extensive compound screening initiatives aiming to discover novel senolytics for the treatment of CKD on a large scale.

## Results

### Induction of cellular senescence in conditionally immortalized human kidney CD10^+^ PTECs and PDGFRβ^+^ fibroblasts

**Fig. 1:**
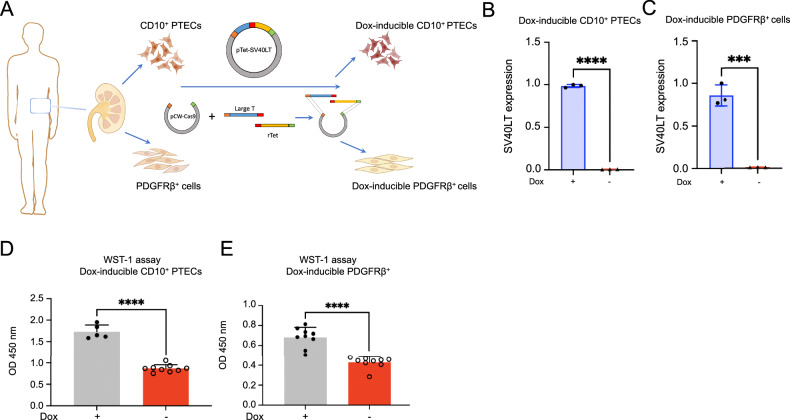
Generation of doxycycline (Dox)-inducible SV40LT in human kidney CD10^+^ proximal tubule epithelial cells (PTECs) and PDGFRβ^+^ fibroblasts. **A** Schematic representation of the generation of dox-inducible SV40LT in cell lines from human kidneys using the pTet-SV40 vector. Figure was created with BioRender. **B**, **C** Relative gene expression of SV40LT in CD10^+^ PTECs and PDGFRβ^+^ cell lines treated with or without doxycycline for 7 days. ***P < 0.001, ****P < 0.0001, *n* = 3. Gene expression was measured using RT-qPCR and visualized as the mean fold change. *P* values were determined by a two-tailed unpaired *t* test. **D**, **E** WST-1 assay in both cell lines treated with or without doxycycline for 7 days; *n* = 5–9. ****P < 0.0001, two-tailed unpaired *t* test. Mean values are shown ± SD.

Conditionally immortalized human kidney CD10^+^ PTECs and PDGFRβ^+^ fibroblast lines were generated using the pTet-SV40LT vector (Fig. 1A, Supplementary Fig. 1A). SV40LT mRNA expression was tightly controlled by doxycycline in both cell lines (Fig. 1B, C). As expected, the growth of CD10^+^ PTECs and PDGFRβ^+^ cells was attenuated by the removal of doxycycline, while the resupplementation of doxycycline increased the expression of SVLT40 and proliferation (Supplementary Fig. 1B, C). This observation indicated that the block of proliferation was reversible in our cell lines. These findings were supported by the results of the cell proliferation WST-1 assay (Fig. 1D, E).

**Fig. 2:**
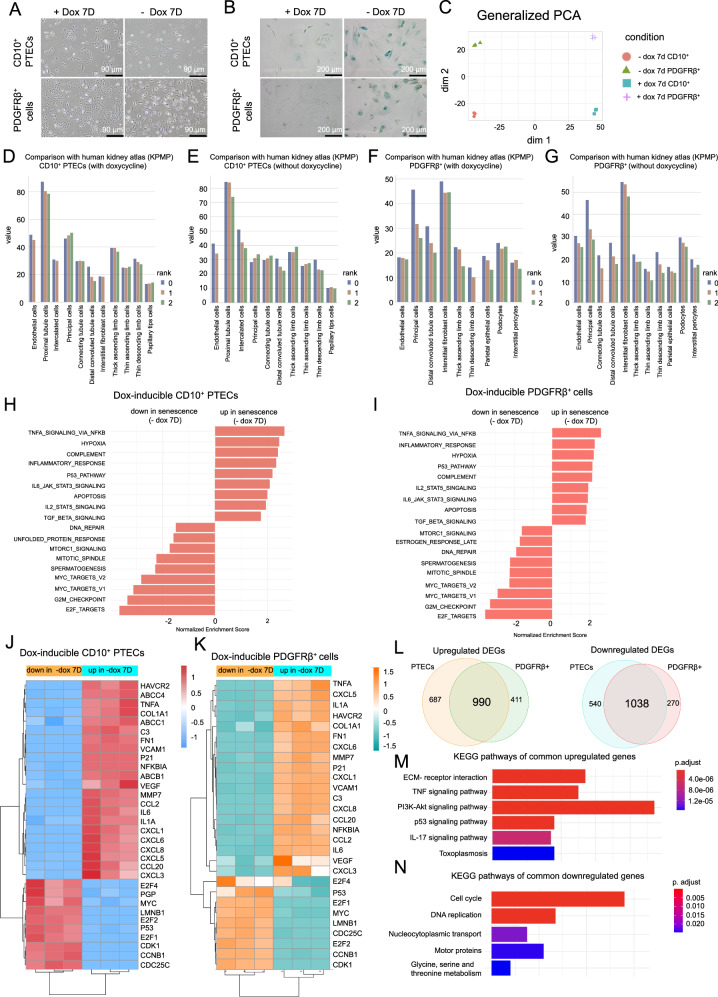
The cell lines matched the respective cell types according to the human single-cell RNA sequencing data and exhibited senescent signatures upon doxycycline withdrawal. Representative phase-contrast microscopy images (**A**) and SA-β-gal staining (**B**) of both cell lines treated with or without doxycycline for 7 days. Scale bars, 90 μm in (**A**) and 200 μm in (**B**). *n* = 4. **C** PCA dimensions of both cell lines treated with and without doxycycline for 7 days. The data were obtained from bulk RNA-seq. **D**–**G** Running mean expression (window size = 10) of the top 3 ranked overlapping marker genes for each cell type is shown for CD10^+^ PTECs and for PDGFRβ+ cells, with and without doxycycline. Irrespective of the doxycycline in the cell culture medium, the KPMP data of CD10^+^ PTECs and PDGFRβ+ fibroblasts showed the highest overlap with human proximal tubules and fibroblasts, respectively. **H**, **I** Hallmark pathways associated with the normalized enrichment score (NES) determined via gene set enrichment analysis (GSEA) of CD10^+^ PTECs and PDGFRβ^+^ cells treated with or without doxycycline for 7 days. A normalized score greater than or less than 0 represented positively or negatively associated pathways, respectively. *n* = 3 each. *P ≤ 0.05. **J**, **K** Heatmap showing the differentially regulated genes (adjusted *p* values ≤ 0.05 and log2-fold change >1 or < 1). Color scales represent after-scaling expression data. Venn diagram (**L**) and KEGG pathway analysis (**M**, **N**) of common up- and downregulated DEGs and pathways across both cell lines.

This cell model allows the expansion of cells far beyond their regular life span through conditional immortalization. As we chose aged individuals to generate these cell lines (85 years old for PTECs and 74 years old for PDGFRb^+^ fibroblasts), we hypothesized that withdrawal of the immortalizing factor would return the aged cells to a primary state of cellular senescence characterized by shortened telomeres, SA-ß-galactosidase activity, and other relevant features. Indeed, when we measured telomere length in both cell lines (Supplementary Fig. 2), we found critically shortened telomeres, nearing the Hayflick limit, which is the threshold at which a cell can no longer undergo further cell division. Furthermore, by removing doxycycline from the cell culture medium, we were also able to induce cellular senescence in both cell lines, as evidenced by increased SA-β-gal activity in enlarged cells (Fig. 2A, B).

### Bulk transcriptome analysis of conditionally immortalized human kidney CD10^+^ PTECs and PDGFRβ^+^ fibroblasts

We next performed bulk RNA sequencing (RNA-seq) to dissect mRNA expression alterations in both cell lines in response to doxycycline withdrawal, placing them in a sudden senescent cell state. Principal component analysis (PCA) indicated that the replicates clustered together with strong differences based on cell identity (epithelial vs. mesenchymal) and a strong effect of senescence (doxycycline vs. doxycycline withdrawal; Fig. 2C). On the basis of gene expression, our cell lines displayed notable resemblances to their in vivo counterparts based on comparisons with publicly accessible human kidney single-cell data from the Kidney Precision Medicine Project (KPMP) (Fig. 2D–G). Thus, our CD10^+^ PTECs were best matched with proximal tubule cells according to the KPMP data (Fig. 2D, E). Similarly, for PDGFRβ+ cells, the best matching cell type according to the KPMP data was interstitial fibroblasts (Fig. 2F, G).

Next, we assessed the pathways associated with doxycycline withdrawal in our cell lines. Gene set enrichment analysis (Hallmarks) revealed enrichment of NF-κB, hypoxia, complement, inflammatory response, P53, IL-2 and IL-6-STAT signaling in senescent cells (-dox 7D) compared to nonsenescent cells (Fig. 2H, I). In contrast, pathways related to cell proliferation and DNA replication were downregulated in senescent cells (Fig. 2H, I). The expression of the senescence marker gene *CDKN1A* (p21) was significantly upregulated in both cell lines upon doxycycline withdrawal, while Lamin B1 expression was downregulated (Fig. 2J, K, Supplementary Table S2). In addition, upon doxycycline withdrawal, the expression of genes related to inflammation (*IL1A, IL6* and TNFα), fibrosis (*VEGF* and *CTGF*), chemotaxis (*CXCL1, CXCL2, CXCL3* and *CXCL8*), and the complement system (*C3*) was markedly increased in both cell lines, while the expression of G2/M and mitosis genes, such as *CDC25C* and *CDK1*, was decreased in senescent cells (Fig. 2J, K). These observations were confirmed by RT‒qPCR (Supplementary Figure 3A‒D). There was a notable convergence of differentially regulated genes (DEGs) in the same direction across both cell lines (Fig. 2L), suggesting a common response in cellular senescence. KEGG pathway analysis of the common DEGs revealed increased TNF-α and p53 signaling in senescent cells, while pathways associated with the cell cycle and DNA replication were downregulated (Fig. 2M, N).

**Fig. 3:**
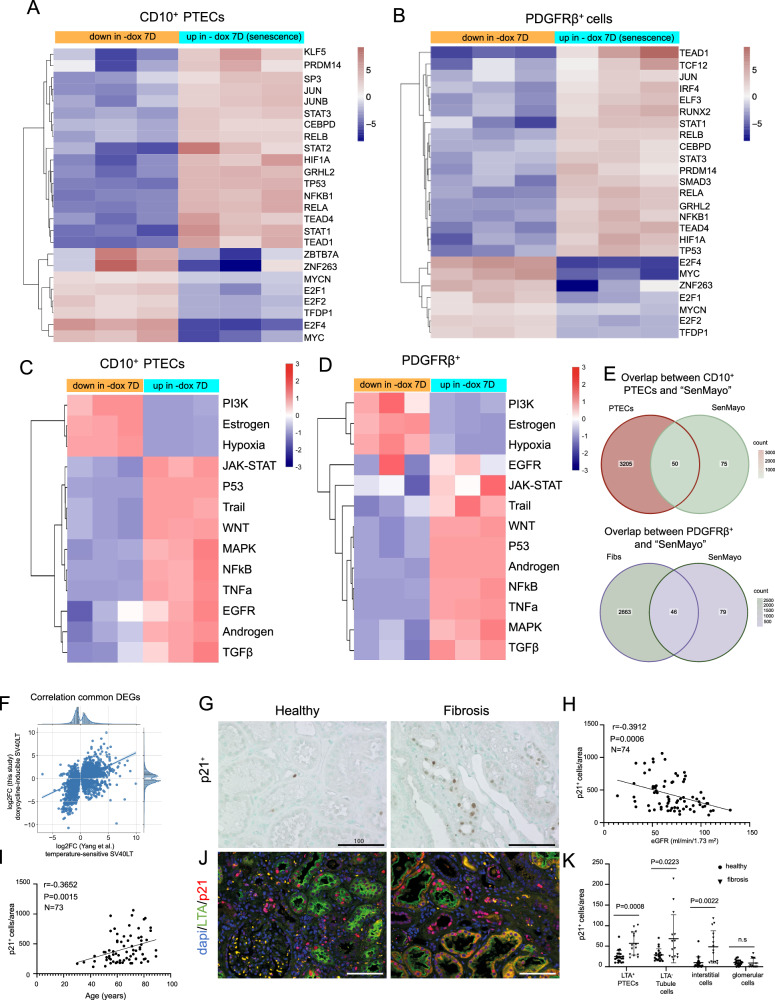
Pathway/transcription factor activities in human kidney CD10^+^ PTECs and PDGFRβ^+^ cell lines with doxycycline-inducible SV40LT expression and staining for p21 in human kidneys. **A**, **B** Transcription factor (TF) activity as estimated by the DoRothEA algorithm across senescent cells (7 days without doxycycline) and nonsenescent cells (7 days with doxycycline) from the human CD10^+^ PTEC and PDGFRβ+ fibroblast bulk RNA-seq dataset. Color scales represent after-scaling expression data. **C**, **D** Pathway activity as estimated by the PROGENy algorithm in senescent cells versus nonsenescent cells using bulk RNA-seq data. Color scales represent after-scaling expression data. **E** Venn diagram of the differentially expressed genes (adjusted *p* ≤ 0.05 and log2-fold change >1 or <1) in senescent CD10^+^ PTECs/PDGFRβ^+^ cells and the “SenMayo” senescence gene set. **F** Comparison of significantly differentially expressed genes (DEGs) (adjusted *p* value < 0.01) between CD10^+^ PTECs treated with doxycycline (nonsenescent) and those not treated with doxycycline (senescent) and between PTECs with temperature-sensitive SV40LT on day 9 at 37 °C (senescent; [15] referred to as day9noIS) and cells at 33 °C on day 0 (immortalized cells, referred to as day0noIS) revealed a Spearman correlation coefficient of 0.54. **G** Immunohistochemical staining of p21 in healthy and fibrotic human kidneys (*n* = 76). Scale bars, 100 μm. **H**, **I** Spearman correlations between the p21^+^ cell population/area and estimated glomerular filtration (*n* = 74) or age (*n* = 73). **J**, **K** Immunofluorescence costaining of p21 and LTA and quantification of the proteins in human kidneys (*n* = 5 for healthy kidney, *n* = 3 for fibrotic kidney). Mean values are shown ± SD.

We used DoRothEA to predict transcription factor activities and found that *JUN*, *TP53*, *RELA, and NFKB1* were increased during senescence in both cell lines (Fig. 3A, B). In addition, increased cell type-specific transcription factor activities were found in PTECs (*SP3, STAT2*, and *KLF5*) and PDGFRβ^+^ cells (*TCF12, RUNX2*, and *IRF4*) (Fig. 3A, B). We also inferred the importance of cell signaling via PROGENy and observed that P53, TGFβ, NF-kB, WNT, and TNF pathway activity increased during the cellular senescence process in both cell lines (Fig. 3C, D). In contrast, the PI3K, estrogen and hypoxia pathways were downregulated in senescent cells. In addition, we found a large overlap with the publicly available SenMayo senescence panel of 125 genes, which has been described to predict aging and senescence across tissues and species (Fig. 3E, Supplementary Table S3) [14]. In summary, these findings suggest that our two novel cell lines can acquire a conserved senescence phenotype.

To rule out the possibility that alterations in gene expression after doxycycline withdrawal result from direct doxycycline effects rather than downstream effects of SV40LT, we compared our PTECs with a previously reported conditionally immortalized PTEC culture model in which SV40LT is regulated by temperature [15] (Fig. 3F). Spearman’s correlation between all common DE genes and statistically significant DE genes (adjusted *p* value < 0.01) was 0.38 and 0.54, respectively. This finding shows a similar signature between our senescent PTECs and previously published PTECs, indicating that the changes observed in our model are unlikely to be attributed to direct effects of doxycycline but rather to the downstream effects of SV40LT degradation. Additionally, we established another CD10^+^ human PTEC line that was permanently immortalized with human telomerase reverse transcriptase (hTERT) and SV40LT. These non-conditionally immortalized cells were then cultured with or without doxycycline to examine the doxycycline-induced changes in the transcriptome (Supplementary Fig. 4A‒C). Compared to the cells treated with doxycycline, the cells without doxycycline exhibited increased gene expression related to coagulation, peroxisome function, cholesterol homeostasis, and proliferation pathways, while pathways associated with p53 and inflammation were downregulated (Supplementary Fig. 4A, B). Therefore, these changes did not reflect those observed in our conditionally immortalized PTECs upon doxycycline withdrawal. This is further emphasized by the poor overlap of DEGs between the two PTEC lines (Supplementary Fig. 4C). Overall, this indicates that the alterations observed in our conditionally immortalized model are not caused by doxycycline itself, but instead result from SV40LT degradation and the onset of senescence.

To assess the significance of these findings within the context of CKD in humans, we analyzed p21 expression in tissue microarrays with kidney cores from 76 patients. Compared to that in healthy kidney tissue, p21 expression was increased in fibrotic tissue and correlated with lower kidney function and higher age (Fig. 3G–I). Coimmunostaining of p21 with the PTEC marker LTA revealed increased p21 expression in LTA^+^ PTECs, LTA^−^ tubular epithelial cells (degenerative PTECs and other tubule segments) and interstitial cells (fibroblasts and immune cells) in fibrosis (Fig. 3J, K**)**. These results suggest that p21 expression may represent a clinically relevant senescence marker in CKD.

### Functional analysis of conditionally immortalized CD10^+^ PTECs and PDGFRβ^+^ fibroblasts

**Fig. 4:**
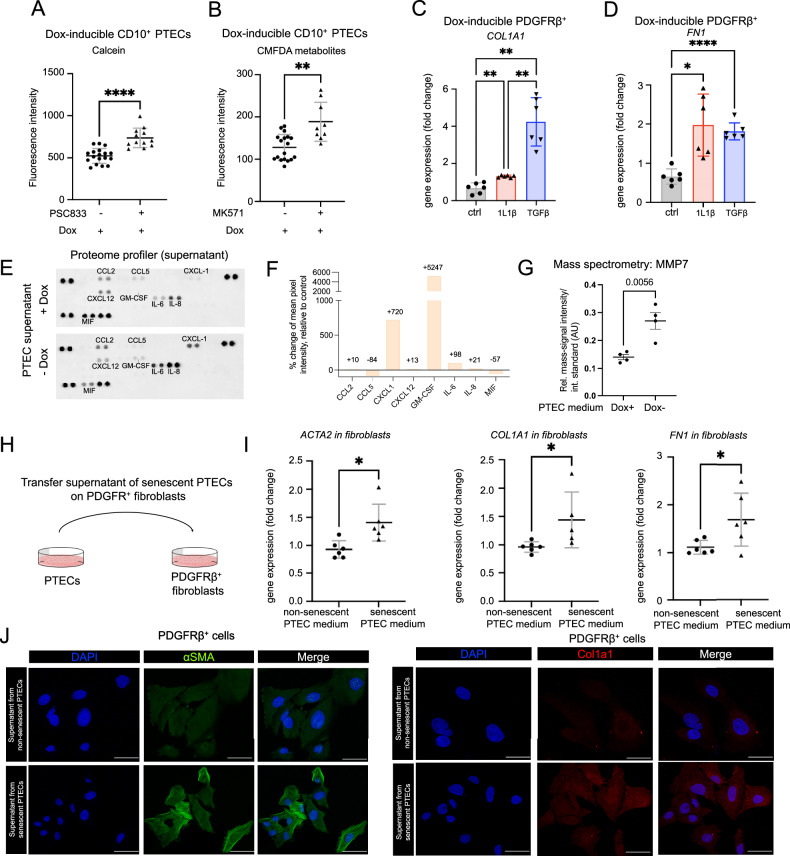
Functional analysis of human kidney CD10^+^ PTECs and PDGFRβ^+^ cell lines with doxycycline-inducible SV40LT expression and crosstalk between PTECs and fibroblasts. **A**, **B** Fluorescence intensity of CD10^+^ PTECs with and without efflux transport blockers. The cells were first incubated with the substrates calcein-AM (1 μM) and CMFDA (1.25 μM), after which the efflux transport blockers PSC833 (5 μM) and MK571 (5 μM) were added. *n* = 9–18, **P < 0.01, ****P < 0.0001, two-tailed unpaired *t* test. **C**, **D** Gene expression of *COL1A1 and FN1* in human kidney PDGFRβ^+^ cells exposed to IL-1β (100 ng/ml) or TGFβ (10 ng/ml). *P ≤ 0.05, **P < 0.01, ****P < 0.0001; two-tailed unpaired *t* test. *N* = 6. **E** Cytokine arrays incubated wi*t*h pooled supernatant from senescent (−dox) and nonsenescent (+dox) PTECs (*n* = 3 per condition was pooled and added to one membrane). **F** Quantification of (**E**). **G** Mass spectrometry signal of MMP-7 in cell culture supernatant from nonsenescent (dox+) and senescent (dox−) PTECs. *N* = 4. **H** Schematic representation of the coculture experiments. **F** Supernatants from day 5 senescent CD10^+^ PTECs were transferred to PDGFRβ^+^ cells for 3 days. **I** Gene expression of *ACTA2*, *COL1A1*, and *FN1* in PDGFRβ+ cells. *P ≤ 0.05, two-tailed unpaired *t*-test. *N* = 5–6. Mean values are shown ± SD. **J** Immunofluorescence staining of αSMA and Col1a1 in PDGFRβ^+^ cells. Scale bars, 50 μm. *N* = 3.

To evaluate whether transporter proteins known to be expressed in proximal tubules [16, 17] are functionally expressed in our human kidney CD10^+^ PTEC line, the efflux protein inhibitors PSC833 and MK571 were used to block the cell transporter activity of P-gp and MRP4, respectively. Using these inhibitors, we detected increased fluorescence intensity of corresponding metabolites of calcein-AM and CMFDA, indicating the presence and activity of these transporters in PT cells (Fig. 4A, B). Based on these findings, we postulate that our PTEC line exhibit functional activity comparable to that of in vivo proximal tubule cells.

Next, we tested whether PDGFRβ^+^ cells respond to increased expression of fibrosis-related genes upon stimulation with the proinflammatory, profibrotic stimuli IL1β and TGFβ. As predicted, RT-qPCR revealed increased expression of typical myofibroblast-related genes (*COL1A1* and *FN1*) after IL-1β and TGFβ stimulation compared to that in vehicle-treated cells (Fig. 4C, D). One hypothesis of kidney fibrosis is that cytokines related to the SASP in senescent PTECs induce fibrosis through the paracrine stimulation of PDGFRβ+. As our transcriptome data revealed the increased expression of several proinflammatory and profibrotic cytokines in senescent cells, we assessed its relative expression on the protein level using a cytokine array kit (Fig. 4E, F). As predicted from our transcriptome data, the supernatant of senescent PTECs showed a relative increase of CCL2, CXCL1, GM-CSF, IL-6 and IL-8 levels, compared to supernatant of nonsenescent PTECs (Fig. 4E, F). Most of these chemokines have been shown to play a role in kidney senescence and fibrosis [3, 8, 18]. In contrast, CCL5 and MIF levels were lower in supernatant of senescent PTECs compared to supernatant of nonsenescent PTECs. Both CCL5 and MIF play a role promoting inflammation, but also proliferation [19, 20]. While one might expect higher levels of CCL5 and MIF due to its inclusion in the proinflammatory SASP, the reduced expression could also be aligned with the low proliferative state of senescent cells [19, 20].

As our transcriptome data showed increased gene expression of MMP-7 and we recently identified MMP-7, released primarily by PTECs, as biomarker for kidney disease progression [21], we also used mass spectrometry to measure MMP-7 levels in the cell culture supernatant of PTECs. This revealed increased expression of MMP-7 levels upon the induction of senescence, which is consistent with a previous study reporting increased expression of MMP-7 in aging kidneys [22] (Fig. 4G).

Next, we transferred the supernatant from our senescent CD10^+^ PTECs to PDGFRβ^+^ fibroblasts cultured with doxycycline (Fig. 4H). The supernatant was mixed with fresh medium at a 1:1 ratio. Control fibroblasts received supernatant from CD10^+^ PTECs cultured in doxycycline-containing medium. RT‒qPCR revealed increased expression of myofibroblast differentiation/fibroblast activation marker genes (*ACTA2, COL1A1, and FN1*) in PDGFRβ^+^ cells cultured in medium from senescent PTECs compared to fibroblasts cultured in medium from nonsenescent PTECs (Fig. 4I). Immunofluorescence staining of PDGFRβ+ cells for alpha smooth muscle actin (αSMA, encoded by ACTA2) and collagen 1 alpha 1 (Col1a1) further supported these mRNA expression results at the protein level (Fig. 4J). Thus, here, we provide proof that senescent human PTECs can drive fibroblast activation and myofibroblast differentiation in a paracrine manner through the SASP.

## Discussion

In this study, we isolated and conditionally immortalized human PTECs and kidney fibroblasts with doxycycline-inducible SV40LT expression. By comparing the gene expression data with human kidney single-cell RNA sequencing data, we showed that these cell lines retained substantial similarity to their in vivo counterparts. We demonstrated that this model enables reversible immortalization of cells for expansion, while withdrawal of the immortalization factor immediately initiates a senescent cell state characterized by cell-cycle arrest and key hallmarks of senescence. The obtained gene set exhibited significant overlap with the publicly accessible SenMayo panel comprising 125 senescence-related genes [14] recognized for their expression during both aging and senescence across various tissues and species. Furthermore, we provided proof that senescent PTECs drive fibroblast activation through paracrine actions. Overall, this conditionally immortalized cell culture model can be employed for the examination of cellular senescence and may serve as a potential platform for screening senolytic drugs to restore kidney function.

Primary cells have a limited capacity for division in culture, primarily due to the progressive shortening of telomeres with each cell division. By expanding and subsequently withdrawing the immortalizing expansion stimulus, we observed that the cells were propelled into a future state marked by shortened telomeres, triggering the onset of cellular senescence. SV40LT [23] or hTERT [24, 25] are commonly used to overexpress SV40LT in immortalized cells. In our pursuit of conditional immortalization, we reasoned that the immortalizing factor must remain entirely inactive when uninduced and should be capable of rapid and uniform induction to attain sufficient expression levels in all cells. Previous studies used temperature-sensitive SV40LT to generate conditionally immortalized glomerular endothelial cells [26], podocytes [27] or proximal tubule cells [28]. In these models, cells proliferate at a permissive temperature of 33 °C but cease growth at 37–39 °C. However, this temperature shift can influence various cellular functions and is not particularly practical. In addition, a large T often does not degrade entirely at increased temperatures, necessitating even higher temperatures, which affect various cellular processes. Here, we used an alternative approach and controlled cell proliferation or arrest by tightly controlling the doxycycline-inducible expression of SV40LT at a consistent temperature of 37 °C. We showed that our system efficiently regulates the expression of SV40LT: in the absence of doxycycline, no SV40LT expression was observed, while doxycycline administration caused robust SV40LT expression with dramatic changes in cell proliferation, morphology and gene expression. Importantly, the observed alterations were a result of the downstream effects of SV40LT and not attributable to doxycycline itself. This was evident in the comparison with temperature-sensitive SV40LT-expressing PTECs, which exhibited similar transcriptomic changes upon induction of senescence.

Both PTECs and fibroblasts are two key players in kidney senescence and fibrosis [29]. By using bulk RNA sequencing, we were able to reveal the senescence gene sets in PTECs and fibroblasts. In the absence of doxycycline, both cell lines expressed common senescence markers and exhibited senescence-related morphological alterations, consistent with the observations in CKD [30]. In particular, we found robust upregulation of the expression of the *CDKN1A* (p21) gene, which is a main driver involved in cell-cycle arrest during senescence [31]. In contrast, induction of cellular senescence causes a reduction in *LMNB1* expression, a widely recognized senescence feature that is linked to impaired nuclear integrity, resulting in the enlargement and irregular shape of cells [32]. We noted an enrichment of genes known to play a role in vivo in failed proximal tubule cell repair, senescence, inflammation and fibrosis, including *NFKB1, TNF, STAT3, VCAM1* and *RELA* [14, 33]. Another hallmark of cellular senescence is the secretion of cytokines, chemokines, proteinases, and other SASP components, which are involved in several biological processes and contribute to tissue dysfunction in aging and disease [32, 34]. As such, upon senescence induction, we found increased gene expression of the proinflammatory genes *IL1*, *IL6,* and *CCL2* in both cell lines. To a large extent, the SASP is a transcriptional program mediated by nuclear factor-kB (NF-kB), which drives the gene expression of *IL1* and *TNFA* [35, 36]. IL6, IL10, and TGFβ are also upregulated through other pathways, such as the JAK2/STAT3 pathway [37, 38]. We also found increased expression of connective tissue growth factor (CTGF) in senescent cells. CTGF is a key downstream mediator of TGFβ signaling and has been demonstrated to regulate profibrotic effects in the kidney [39, 40].

Cellular senescence is a complex process that involves multiple signaling pathways. We found decreased PI3K pathway activity and estrogen and hypoxia pathway activity in senescent cells. PI3K signaling is essential for cell growth and proliferation. It has been shown earlier that PI3K/AMPK/mTOR activity is reduced during the senescence of irritated endothelial cells [41], leading to decreased levels of the downstream targets EIF4E, RPS6, and EIF3 and many ribosomal proteins relevant for cell growth and protein translation. Furthermore, PTEC senescence can be induced by the inhibition of AMPK–mTOR signaling [42]. Similarly, the estrogen pathway promotes cell growth, and recent research has suggested that cellular senescence may underlie sex differences [43]. Estrogen can suppress the release of reactive oxygen species (ROS), thereby preventing ROS-induced DNA damage. In addition, estrogen suppresses p21 expression, SASP factor expression and the JAK/STAT pathway [44] and is important for telomere maintenance and autophagy. Consistent with the findings of previous reports, we also found increased expression of *HIF1A* in senescent cells, which accumulates under hypoxic conditions and regulates cellular senescence by affecting p21, p53, and lamin B1 levels [45]. In contrast to these three pathways, we found increased activity of several pathways (JAK-STAT, p53, MAPK, NF-kB, TNFalpha, TGFbeta, and Wnt), which are involved in senescence [31]. Overall, our results indicate that our conditional cell culture model can recapitulate the hallmarks of cellular senescence.

Interactions between epithelial cells and mesenchymal components, specifically fibroblasts and pericytes, have been recognized as hallmarks of CKD [7]. In nearly every instance of kidney injury, PTECs sustain damage, whether secondary to glomerular injury-induced proteinuria or direct toxic or hypoxic injury. Consequently, we propose that the induction of a senescent phenotype in PTECs could lead to the subsequent activation of fibroblasts through the SASP. There is some evidence from murine models that crosstalk between PTECs and fibroblasts via paracrine actions contributes to the pathophysiological processes associated with cellular senescence and kidney fibrosis [46]. Indeed, we identified several proinflammatory and profibrotic proteins, known to be components of the SASP, in the supernatant of senescent PTECs, and demonstrated that human senescent PTECs drive fibroblast activation in a paracrine manner. Furthermore, the number of senescent PTECs correlated positively with the severity of kidney fibrosis and negatively with kidney function in a human cohort of >70 patients.

In summary, our study offers a novel conditional senescence cell culture model for studying cellular senescence in the human kidney. These cell lines may serve as valuable tools for potential therapeutic target identification and drug testing and provide evidence that paracrine signaling in senescent PTECs might be involved in kidney fibrosis and CKD.

## Materials and methods

### Ethics and Human Kidney Tissue Collection

The present study was approved by the local ethics committee of the University Hospital RWTH Aachen (EK-016/17). Unaffected healthy kidney tissues were obtained from patients who underwent nephrectomy for kidney cancer (distant from any cancer as confirmed by a blinded pathologist). Written informed consent was obtained from all patients, and the study was performed in accordance with the Declaration of Helsinki. The work at biosafety level S2 was approved by the German authorities (reference number 53.05-01-K-21-093).

### Isolation of primary CD10^+^ PTECs and PDGFRβ^+^ fibroblasts

PTECs can be marked as clusters of 10 differentiated (CD10^+^ cells), and our recent single-cell atlas of human kidney fibrosis suggested that PDGFRβ^+^ fibroblasts are the main cellular source of scar-forming myofibroblasts [5]. Thus, in this study, we sorted CD10^+^ PTECs and PDGFRβ^+^ fibroblasts as described below. We aimed to include kidney tissue from individuals over 65 years old with a eGFR of less than 60 ml/min/1.73 m². These inclusion criteria were chosen to isolate cells with already shortened telomeres due to aging and CKD.

Kidney tissue (85 years old, female, eGFR: 30 ml/min/1.73 m^2^) was mechanically dissociated into 0.5–1 mm^3^ pieces, and a single-cell suspension was obtained by using gentle-MACS (Miltenyi Biotec, Bergisch Gladbach, Germany) as described previously [5]. The cells were first incubated with Fc-Block (Trustain anti-human, 1:50; Biolegend, Cat#422302; RRID: AB_2818986) for 30 min and then stained with anti-CD10 (clone HI10a, 1:40; Biolegend Cat#312214; RRID: AB_2146548), anti-CD45 (clone HI30, 1:40; Biolegend Cat#304006; RRID: AB_314394), and anti-CD31 (clone WM59, 1:40; Biolegend, Cat#303106; RRID: AB_314332) antibodies for another 30 min at room temperature. DAPI (#D9542, Sigma) was used for dead cell staining. CD10^+^/CD31^−^/CD45^−^/dapi^-^ PTECs were sorted using a SONY SH800 sorter (Sony Biotechnology, San Jose, CA, USA) (Supplementary Fig. S1).

To isolate PDGFRβ^+^ fibroblasts from the kidney (74 years old, male, eGFR: 59 ml/min/1.73 m^2^), single-cell suspensions were subjected to magnetic-activated cell sorting (MACS) according to the manufacturer’s instructions. In brief, cells were stained with a human PDGFRβ^+^ antibody (Clone PR7212, 1:100; R&D Systems, Cat#MAB1263; RRID: AB_2162792), followed by magnetic separation using anti-mouse IgG1 microbeads (Miltenyi Biotec, Cat#130-047-102; RRID: AB_244355) to obtain PDGFRβ^+^ fibroblasts.

Primary CD10^+^ PTECs and PDGFRβ^+^ fibroblasts were cultured in DMEM/F12 and GlutaMAX^TM^ Medium (Gibco Cat# 31331026, Grand Island, New York, USA) supplemented with 10% fetal calf serum (PAA, Cat# A01125-499, Cölbe, Germany) and 100 U/ml penicillin‒streptomycin (Gibco) at 37 °C and 5% CO_2_ with saturating humidity. All cell models were regularly tested for mycoplasma contamination.

### Conditional immortalization of CD10^+^ epithelial cells and PDGFRβ^+^ fibroblasts

Primary CD10^+^ PTECs and PDGFRβ^+^ fibroblasts were immortalized via lentiviral infection with SV40LT. Here, we generated a doxycycline-inducible lentiviral vector (pTet-SV40LT-Puro) expressing SV40LT using a Gibson assembly kit (Cat#E5510S, NEB, Frankfurt, Germany; Gibson et al., 2009). In brief, pCW-Cas9 (Cat# 50661, Addgene) [47] was opened using NheI (Cat# R3131V, NEB) and KpnI (Cat #R3142V, NEB) as the backbone. SV40LT was amplified from pBABE-puro SV40LT (Cat #13970, Addgene) [48], and the EF1α-Puro-rTetR-WPRE element was amplified from TLCV2 (Cat #87360, Addgene) [49] by PCR using Q5 High-Fidelity DNA Polymerase (Cat #M0491V, NEB). For each cell line, 0.4 million primary cells were infected with lentiviral particles expressing SV40LT from five 6 cm dishes. HEK293T cells (0.8 million, 70–80% cell confluence) were transected with 5 μg of pTet-SV40LT-Puro, 2.5 μg of psPAX2 (Cat #12260, Addgene) or 2.5 μg of pMD2. G (Cat #12259, Addgene) per 6 cm dish by calcium phosphate precipitation [50]. The virus particles were concentrated by chondroitin sulfate sodium salt (CSS) and polybrene precipitation [51] as described previously [52]. Primary CD10^+^ PTECs and PDGFRβ^+^ fibroblasts were infected with SV40LT lentiviral particles for 4 h, after which the medium was replaced with complete growth medium supplemented with 10 μg/ml doxycycline (Cat #D9891, Sigma). The CSS precipitates were removed by daily washing with PBS, and the medium was changed. Two days after infection, 2 μg/ml puromycin (Cat #P8833, Sigma‒Aldrich) was used to select SV40LT-expressing cells for 7 days, and doxycycline was added to maintain SV40LT expression and maintain cell immortalization.

### Measurement of telomere length using flow FISH

Fluorescence in situ hybridization (flow-FISH) was carried out as described previously [53]. Briefly, vital sterile frozen PTECs and fibroblasts treated with or without doxycycline were subjected to flow-FISH analysis of telomere length. Samples were prepared for cell denaturation and mixed with a fluorescein isothiocyanate (FITC)-labeled telomere-specific (CCCTAA) 3-peptide nucleic acid FISH probe (Eurogentec, Liège, Belgium) for DNA hybridization, followed by DNA counterstaining with LDS 751 (Sigma). Bovine thymocytes were used as internal controls. The predeterminate telomere length of bovine thymocytes was used to calculate the length in kilobases within PTECs and fibroblasts (Supplementary Fig. 2).

### Cell proliferation assay

Cell proliferation was monitored using a WST-1 assay (Cat #5015944001, Merck) according to the manufacturer’s instructions. In brief, 2 × 10^3^ CD10^+^ or PDGFRβ^+^ cells were seeded into a 96-well plate with or without doxycycline. The cells were incubated with WST-1 reagent for 2 h at 37 °C and 5% CO_2_ on day 7. The OD value was then determined using a spectrometer (CLARIOstar, Ortenberg, Germany) at 450–600 nm absorbance.

### Senescence assay

Senescence β-galactosidase (SA-β-Gal; Cat#9860S; Cell Signaling Technology, Boston, MA, USA) was used to detect SA-β-gal in senescent cells. In brief, 2 × 10^4^ CD10^+^ PTECs or PDGFRβ^+^ cells were cultured in 12-well plates with or without doxycycline for 7 days and then stained with SA-β-Gal according to the manufacturer’s instructions. Images were acquired with an optical microscope (ZEISS1846, Oberkochen, Germany).

### PDGFRβ^+^ fibroblast stimulation with IL-1β and TGFβ

Immortalized PDGFRβ^+^ fibroblasts at 70% confluence were starved for 24 h in DMEM containing 0.5% FCS. Then, IL1β (100 ng/ml; Cat# SRP3083, Sigma‒Aldrich) and TGFβ (10 ng/ml; Cat #100-21, PeproTech, Hamburg, Germany) were added to the medium. The medium was changed after 24 h, and the cells were harvested after 48 h of treatment.

### Doxycycline treatment of non-conditionally immortalized CD10+ human proximal tubule cells

CD10^+^ PTECs were isolated from the healthy kidney cortex of a 55-year-old male nephrectomy specimen. To isolate PT cells, a single-cell suspension was prepared as described above, incubated with CD10-Microbeads (Miltenyi Biotec, #130-093-452), and separated using MACS technology (Miltenyi Biotec, autoMACS Pro Separator, #130-092-545, autoMACS Columns #130-021-101). The isolated primary PTECs were cultured in DMEM/F12 (1:1) with Glutamax (Gibco, #31331), supplemented with 10% FCS and 1% Pen/Strep for three weeks. To immortalize the PT cells, retroviral particles SV40LT and HTERT were employed. Retroviral particles were produced by transient transfection of HEK293T cells using TransIT-LT (Mirus). Two types of amphotropic particles were generated by co-transfecting plasmids pBABE-puro-SV40-LT (Addgene 13970) or xlox-dNGFR-TERT (Addgene 69805) along with a packaging plasmid pUMVC (Addgene #8449) and a pseudotyping plasmid pMD2.G (Addgene 12259). Retroviral particles were concentrated 100-fold using Retro-X concentrator (Clontech) 48 h post transfection. Cell transduction was performed by incubating the target cells with serial dilutions of the retroviral supernatants (a 1:1 mix of concentrated particles containing SV40-LT or hTERT) for 48 h. Subsequently, the infected PT cells were selected using 2 μg/mL puromycin 72 h after transduction for 7 days. These immortalized CD10^+^ PT cells were then cultured in 10 μg/ml doxycycline (Cat #D9891, Sigma) for 7 days to investigate the doxycycline-induced changes in the transcriptome.

### RT-qPCR assay

Total RNA was isolated using a RNeasy Mini Kit (Cat# 74106; Qiagen, Hilden, Germany) according to the manufacturer’s instructions. The RNA concentration was measured with a Nanodrop 2000 (Thermo Fisher, Waltham, MA, USA). Total RNA (1 μg) was reverse transcribed with a High-Capacity cDNA Reverse Transcription Kit (Cat# 43-688-13; Applied Biosystems, Foster, CA, USA). A real-time PCR system (Applied Biosystems) was used to perform RT-qPCR using SYBR Green fluorescence (Cat# 1725125, Bio-Rad, Feldkirchen, Germany). The primers used for RT-qPCR are listed in Supplementary Table 1. Human *GAPDH* was used for normalization of gene expression.

### Bulk RNA sequencing and data analysis

RNA was isolated as described above. rRNA was depleted within 200 ng of total RNA, and sequencing libraries were generated using the NEBNext Ultra II Directional RNA library kit (Cat#E7760L, NEB) according to the manufacturer’s protocol. In the initial preprocessing step, nextflow workflows (version 21.04.1) [54] were applied, specifically the nf-core/rnaseq (version 3.1) pipeline [55]. This involved the use of STAR (version 2.7.9a) [56] for read alignment, Salmon (version 1.5.0) [57] for read quantification, TrimGalore (version 0.6.6) for read trimming, and GENCODE (version 38) for gene annotation [57]. Subsequently, the generated count matrix file from Salmon was filtered, excluding genes labeled ‘Mt_tRNA,’ ‘rRNA,’ ‘Mt_rRNA,’ and ‘rRNA_pseudogene’ in the GENCODE annotation file. Additionally, genes associated with low expression were removed using HTSFilter (version 1.32.0) [58]. In the final step, DESeq2 (version 1.32.0) [59] was used to identify DEGs from the filtered count matrix file for the specified comparisons. PCA was performed by using the glmpca package [60]. For hierarchical clustering, the distance between each sample was calculated by the “dist” function with the “average” method in R. Afterwards, all the samples were clustered by “hclust” and visualized by the “gplots” package. The DEGs were ranked by the Wald statistic for pathway analysis. P values were adjusted for multiple testing using the Benjamini and Hochberg method. Genes and pathways with an FDR < 0.05 were considered significant. We used GSEA-preranked to test for enrichment of senescence genes in the phenotypes using the fgsea R package (v.1.14.0) [61]. In the following heatmaps, a subset of significant genes was displayed with the “Pheatmap” R package. The PROGENy tool was used to estimate pathway activity in senescent and nonsenescent cells [62]. The DoRothEa database was used to predict transcription factor-binding sites and infer regulatory networks [63, 64]. Venn diagrams were generated with the “VennDiagram” package in R (version 4.2.1). The upregulated (log fold change ≥1 and adjusted *p* value ≤ 0.05) and downregulated (log fold change ≤ 1 and adjusted *p* value ≤ 0.05) genes in both CD10^+^ PTECs and PDGFRβ^+^ cells were chosen as the intersections in the Venn diagrams. The “ClusterProfiler” package in R (version 4.2.1) was used to perform the KEGG pathway enrichment analysis based on the 990 common upregulated and 1038 common downregulated genes. Pathways with adjusted *p* values < 0.05 were considered significant. Furthermore, the overlap between the previously published panel of 125 senescence genes [13] and our two cell lines was compared.

### Cell type comparison with publicly available human kidney single-cell transcriptomics data

Single-cell transcriptomics can be used to understand the cellular heterogeneity of complex biological systems such as the kidney. To estimate the similarity of our cell lines to their relevant cell types in vivo, we used publicly available single-cell transcriptomics data from the KPMP consortium (atlas.kpmp.org/repository). Briefly, overlapping differentially expressed genes were identified for each cell line and cell type in the single-cell data and ranked based on their average gene expression in that cell type. The cell types with the highest average gene expression were considered the best matches to our cells. To this end, KPMP cell type annotation data consisting of 200338 single cells were obtained from KPMP.org. The raw count data were log-normalized using the normalize_total and log1p functions in Scanpy (version 1.9.2), and the marker genes for each cell type were calculated using the Wilcoxon method implemented in the rank_genes_groups function. Marker genes with adjusted *p* values < 0.01 were used for downstream analyses. The overlap of these marker genes with the top 100 DE genes (logFC > 0 and adjusted *p* value < 0.05) for each cell line (CD10^+^ PTECs vs PDGFRβ+ fibroblasts) obtained using DESeq2 was then used to identify the common genes. The running average (window size = 10) of the top overlapping genes with respect to the log2-fold change was then calculated for each cell type in the single-cell data.

### Correlation with the publicly available conditionally immortalized PTEC model

To rule out the possibility that alterations in gene expression after doxycycline withdrawal result from direct doxycycline effects rather than downstream effects of SV40LT degradation, we compared our PTECs with a previously reported conditionally immortalized PTEC culture model (ciPTEC-OAT1) in which SV40LT expression is regulated by temperature [15]. In this model, p53 and p21 are activated at a permissive temperature (33 °C) through the expression of the temperature-sensitive mutant U19tsA58 of the SV40 large T antigen (SV40T). Moreover, the cells exhibit senescence-like arrest upon shifting to a nonpermissive temperature (37 °C). In their study, cells were cultured until 90% confluence at 33 °C and then transferred to 37 °C with 5% (v/v) CO_2_ to allow them to mature for 0, 1, 3 or 9 days. For comparison with our RNA-seq data, we used day 0 at 33°C and day 9 at 37 °C, both without indoxyl sulfate treatment. To compare the dysregulated genes in the two cell lines, we calculated the differentially expressed genes in our CD10+ PTECs (doxycycline negative vs doxycycline treated) and proximal tubule cells (senescent vs immortalized) obtained from [15].

### Fluorescence-based functional assays of CD10^+^ PTECs

The functional activities of the ATP-dependent transporter P-glycoprotein (P-gp) and the organic anion transporter human multidrug resistance protein 4 (MRP4) in CD10^+^ PTECs were evaluated using fluorescent substrates that accumulate intracellularly upon the inhibition of efflux transporters. Calcein-AM (Cat# 425201; BioLegend, San Diego, CA, USA) and CMFDA (5-Chlormethylfluorescein diacetate) (Cat# C7025; Thermo Fisher Scientific, Eugene, OR, USA) were used to evaluate transporter activity. Despite the significant substrate overlap among the transporters, their distinct binding properties enable different substrate specificities [65]. Calcein-AM and CMFDA acquire fluorescent characteristics following hydrolysis (cleavage of methyl groups) in the cytoplasm. In the case of CMFDA, this process is followed by conjugation to carboxyfluorescein-glutathione, involving the replacement of the chlorine group. These resultant fluorescent metabolites were utilized to assess the activities of P-gp (calcein) and MRPs (CMFDA metabolites), respectively. The inhibitors MK571 (Cat# HY-19989A, MedChem Express, Monmouth Junction, NJ, USA) and PSC833 (Cat# HY-17384, MedChem Express) were used as known inhibitors of MRP4 and P-gp, respectively. The dilutions of the different inhibitors were varied starting at a concentration of 5 μM. To test for CMFDA metabolites retention, the cells were incubated with 1.25 μM CMFDA in combination with 5 μM MK571 for 30 min with doxycycline. Subsequently, the cells were washed with Krebs–Henseleit buffer (Cat# K3753, Merck), and fluorescence was immediately measured (wavelengths: excitation at 492 nm; emission at 517 nm). Calcein retention was assessed by incubating the cells with 1 μM calcein-AM for 1 h in combination with the other inhibitor. Afterward, the cells were washed with Krebs–Henseleit buffer and lysed with 1% Triton X-100 (Cat#T8787, Sigma‒Aldrich). Fluorescence was measured after 45 min (wavelengths: excitation at 485 nm; emission at 530 nm). The pH of the Krebs–Henseleit buffer was maintained at 7.4, and fluorescence readings were performed using a CLARIOstar microplate reader (BMG LABTECH). All tests were performed in 96-well plates, three times in at least three independent experiments.

### PTEC-Fibroblast crosstalk experiments

Supernatants from day 5 CD10^+^ PTECs not treated with doxycycline were mixed with fresh medium at a ratio of 1 to 1 and were referred to as senescent medium. The PDGFRβ^+^ cells were cultured for 3 days with doxycycline and senescent medium and then harvested. Control PDGFRβ^+^ cells received supernatant from PTECs that were cultured with doxycycline in the medium.

### Cytokine array

The relative levels of various secreted cytokines were measured in the cell culture supernatants of conditionally immortalized PTECs using a cytokine array (Proteome Profiler Human Cytokine Array Kit, R&D Systems, Cat# ARY005B). PTECs were plated on 6-well plates at a density of 60,000 cells/well in 2 ml/well medium, respectively. Seven days after plating, with and without doxycycline treatment, cell supernatants were collected, pooled for each condition (*n* = 3), and centrifuged twice (1600 rpm, 5 min). The relative cytokine levels were visualized according to the manufacturer’s instructions, using 0.5 ml of the supernatant. The membranes were simultaneously imaged using the Chemi-Doc MP system (Bio-Rad) with a consistent exposure time. The mean pixel intensities, normalized to the internal control spots, were quantified using ImageJ2 software (version 2.14).

### Quantification of MMP-7 using matrix-assisted laser desorption/ionization (MALDI)–time-of-flight mass spectrometry

Mass spectrometry was utilized to measure MMP-7 levels since it was not included in the cytokine array kit. Twenty micrograms of protein from the supernatant of PTECs with or without doxycycline was separated by SDS-polyacrylamide gel electrophoresis. Proteins were stained using Coomassie Brilliant Blue G-250 (BioRad, Munich, Germany). The gel plugs were manually excised and then washed and equilibrated using ammonium bicarbonate in acetonitrile. The isolated protein was digested with trypsin and analyzed by matrix-assisted laser desorption/ionization-time-of-flight mass spectrometry (MALDI-TOF-TOF) as previously described [66, 67]. A database search (Swiss-Prot) was performed using the Mascot 2.2 search engine (Matrix Science, Boston, MA) and Bruker Bio-Tool 3.2 software. The calibrated and annotated spectra were used to calculate the peptide mass signals for each entry in the sequence database, compare the experimental MALDI-MS and MALDI-MS/MS datasets, and assign a statistical weight to each individual peptide match using empirically determined factors.

### Immunofluorescence

For cultured cells, the following antibodies were used: anti-COL1α1 (1:100; Abcam Cat# ab34710; RRID: AB_731684), anti-actin alpha SMA-FITC (1:100; Sigma Cat# F3777; RRID: AB_476977), and AF555-conjugated anti-rabbit (1:100; Vector Cat# BA-1000; RRID: AB_2313606). PDGFRβ^+^ cells were fixed in 4% formalin for 10 min at room temperature. Triton-X (0.1%) was used to permeabilize the cells for 20 min. The cells were blocked in 2% bovine serum, incubated overnight with a primary antibody, washed with PBS and subsequently incubated with the secondary antibody for 2 h.

For human tissue sections, deparaffinization and antigen retrieval (Cat# H-3300, Vector) were performed as described below. The sections were incubated with blocking buffer (1% donkey serum in PBS) followed by incubation with a p21 antibody (1:100; Cell Signaling Cat#2947; RRID: AB_823586). The sections were washed with PBS and then incubated with Cy3-conjugated secondary antibodies (1:100; Jackson ImmunoResearch Cat# 711-165-152; RRID: AB_2307443) and Lotus Tetragonolobus Lectin LTA (1:200; Vector Cat#FL1321; RRID: AB_2336559) for 60 min. Cells and tissue sections were stained with 4′,6-diamidino-2-phenylindole (DAPI) (1:10,000, Sigma‒Aldrich). Images were acquired with a Nikon A1R confocal microscope. The raw imaging data were processed using Nikon Software and ImageJ.

### Immunohistochemical staining and scanning of tissue microarrays

Microarrays containing cores of human kidney tissue were used for p21 staining. Deparaffinization was achieved by immersing the slides in 100% xylene for three rounds of 5 min each. This was followed by 2 min of immersion in decreasing ethanol concentrations (3 × 100%, 2 × 96%, and 1 × 70%) to rehydrate the tissue, followed by a citrate-based antigen retrieval step. A 10-min step involving the addition of 3% hydrogen peroxide (Cat# 8070.4; Carl Roth, Karlsruhe, Germany) was then carried out to block peroxidase activity. Subsequently, the tissue was washed twice with PBS, after which avidin and biotin were added to the tissue for 10 min each. The tissue was stained with a p21 antibody (1:100; Cell Signaling Cat#2947) at room temperature for 90 min and then washed with PBS, followed by incubation with a biotinylated anti-rabbit antibody (1:100; Vector Cat#BA-1000; RRID: AB_2213606) for 30 min. This was followed by washing with PBS and incubating with the avidin-biotin complex (Vector Cat# SP-2001, RRID: AB_2336231) for 30 min. After two washes, the HRP substrate DAB (Vector Cat# SK-4100, RRID: AB_2336382) was added to the tissue for 10 min. The entire slide was scanned. The image files were imported into QuPath [68], and p21^+^ cells were manually counted in 76 cores. Cores were excluded from the analysis when age (*N* = 3) or estimated glomerular filtration rate (eGFR) (*N* = 2) were missing.

### Statistics

Variance homogeneity was tested using F tests. Additionally, the data were assessed for a normal distribution using the D’Agostino and Pearson test. To compare two groups, an unpaired t test or the Mann‒Whitney test was applied when the data were normally distributed. When comparing more than two groups, one-way analysis of variance (ANOVA) with Brown–Forsythe test was used. The correlation between two variables was calculated using the Spearman correlation coefficient. The ROUT test was employed to identify outliers. A *p* value less than 0.05 was considered to indicate statistical significance. All the experiments were repeated at least three times, and the experimental data were statistically analyzed using GraphPad Prism 9.0. The data are presented as the means ± standard deviations (SDs).

### Supplementary information

Supplemental Figures

Supplemental Data as Excel File

## Data Availability

The authors declare that the processed bulk RNA-seq count matrix data in the article are available at https://zenodo.org/records/10532131. The sequencing data generated in this study will be available in the GEO database with the publication of the manuscript. The code used to analyze the data in this project is provided at https://github.com/hayatlab/conditional_senescence. The KPMP data can be downloaded for use from the KPMP Atlas Repository atlas.kpmp.org/repository. The bulk seq data from the conditionally immortalized human proximal tubule cell line in which SV40LT is controlled by temperature can be found at DOI: 10.3390/toxins15040242. The “SenMayo” senescence gene set is available in supplementary data 1 at 10.1038/s41467-022-32552-1.
