# Supplementary material for: Generation of a conditional cellular senescence model using proximal tubule cells and fibroblasts from human kidneys
Source: Cell Death Discov. 2024 Aug 14;10:364. doi: 10.1038/s41420-024-02131-y (PMC11324798; doi:10.1038/s41420-024-02131-y)
Supplement: Supplementary file 1 — Supplemental Figures [file 41420_2024_2131_MOESM1_ESM.pdf]

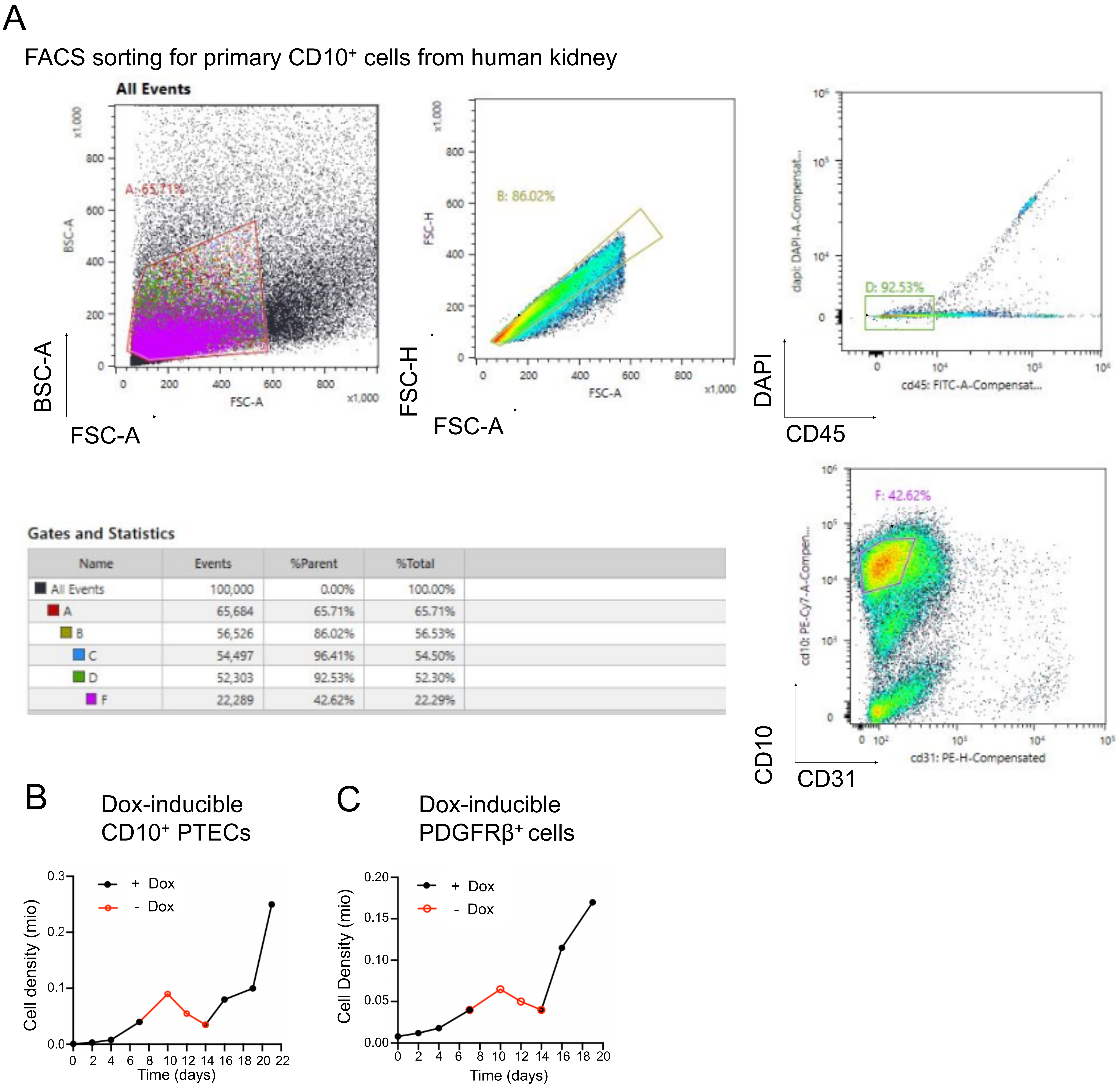

Name	Events	%Parent	%Total
All Events	100,000	0.00%	100.00%
A	65,684	65.71%	65.71%
B	56,526	86.02%	56.53%
C	54,497	96.41%	54.50%
D	52,303	92.53%	52.30%
F	22,289	42.62%	22.29%

Supplemental Figure S1. A: CD10+/CD31-/CD45-/dapi- human proximal tubule cells were sorted. **B-C**: Representative growth curves for both cell lines. Cell numbers in millions (mio) were calculated every 2-3 days. Absolute numbers in one representative experiment per cell line is shown. N=3. Cells were kept from day 7 to 14 without doxycycline in cell culture medium.

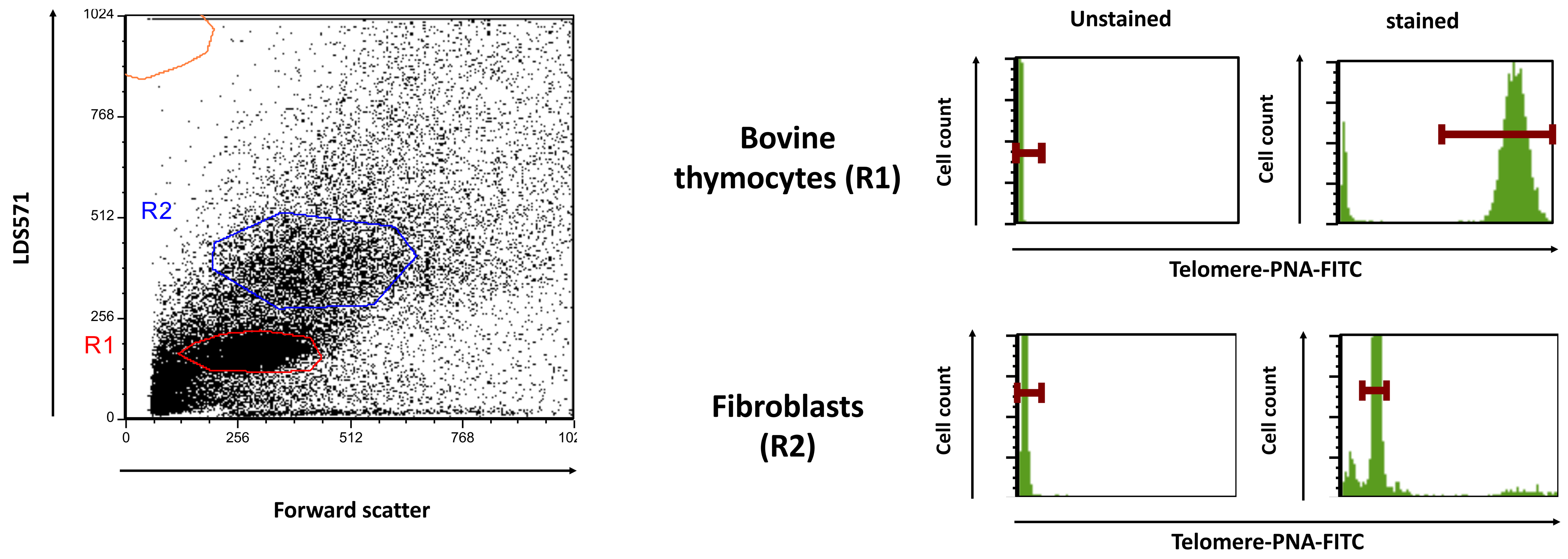

Condition	Median telomere length (kb)
PTECs with doxycycline at day 7	4.4
PTECs without doxycycline at day 7	4.2
PDGFRb$^+$ fibroblasts with doxycycline at day 7	3.7
PDGFRb$^+$ fibroblasts without doxycycline at day 7	3.5

Supplemental Figure S2. Flow-FISH was carried out to measure the telemore length in proximal tububle epithelial cells (PTECs) and fibroblast cells. As an example, telomere length analysis of bovine thymocytes (as control) and human kidney fibroblasts is shown.

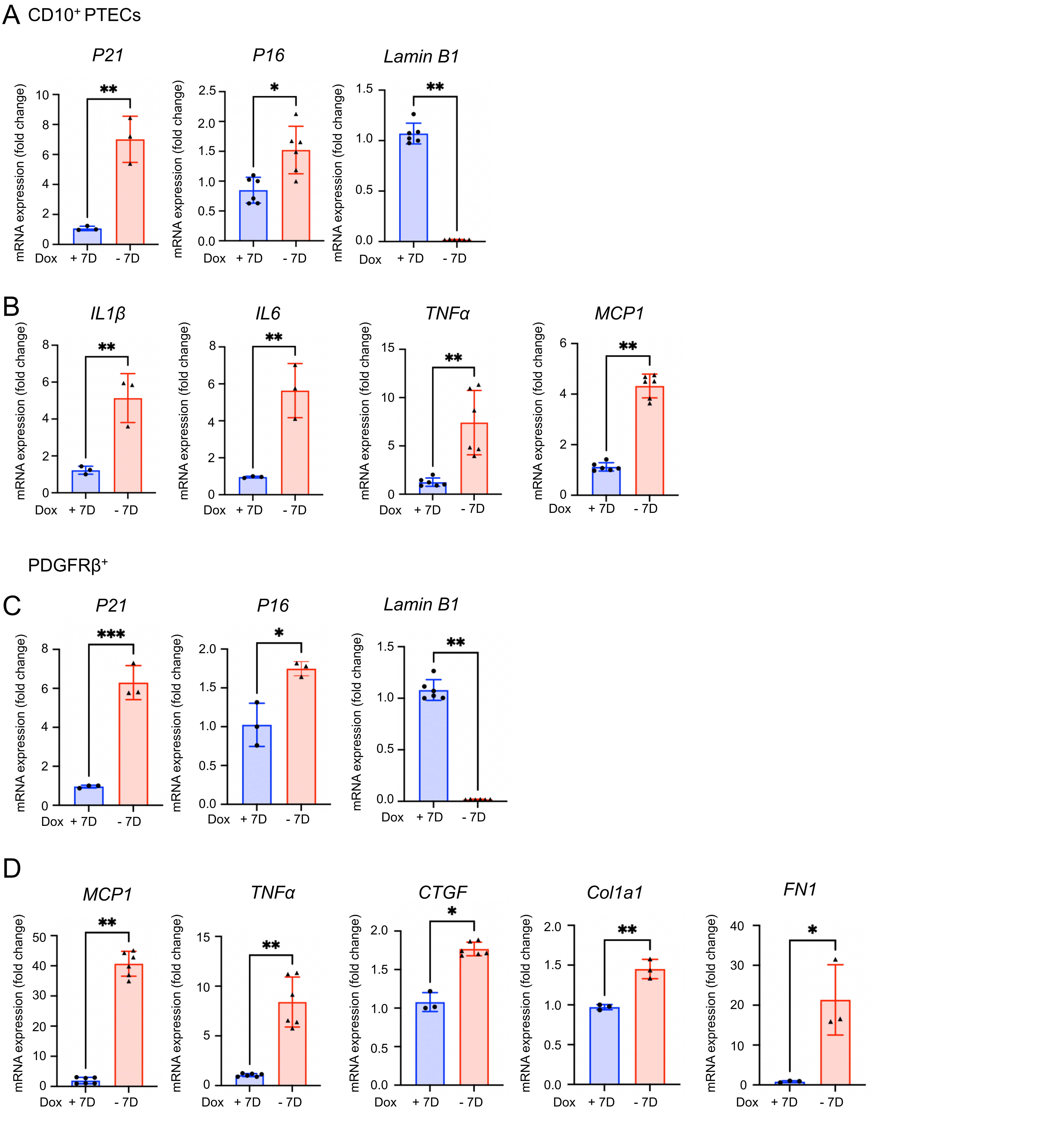

Supplemental Figure S3. Doxycycline withdrawal induced expression of senescence-associated genes in CD10+ PTECs (A-B) and PDGFRβ+ cells (C-D), respectively. n=3-6, *$P < 0.05$, **$P < 0.01$, *** $P < 0.001$, two-tailed unpaired *t*-test. N=3-6. Mean values are shown ± SD.

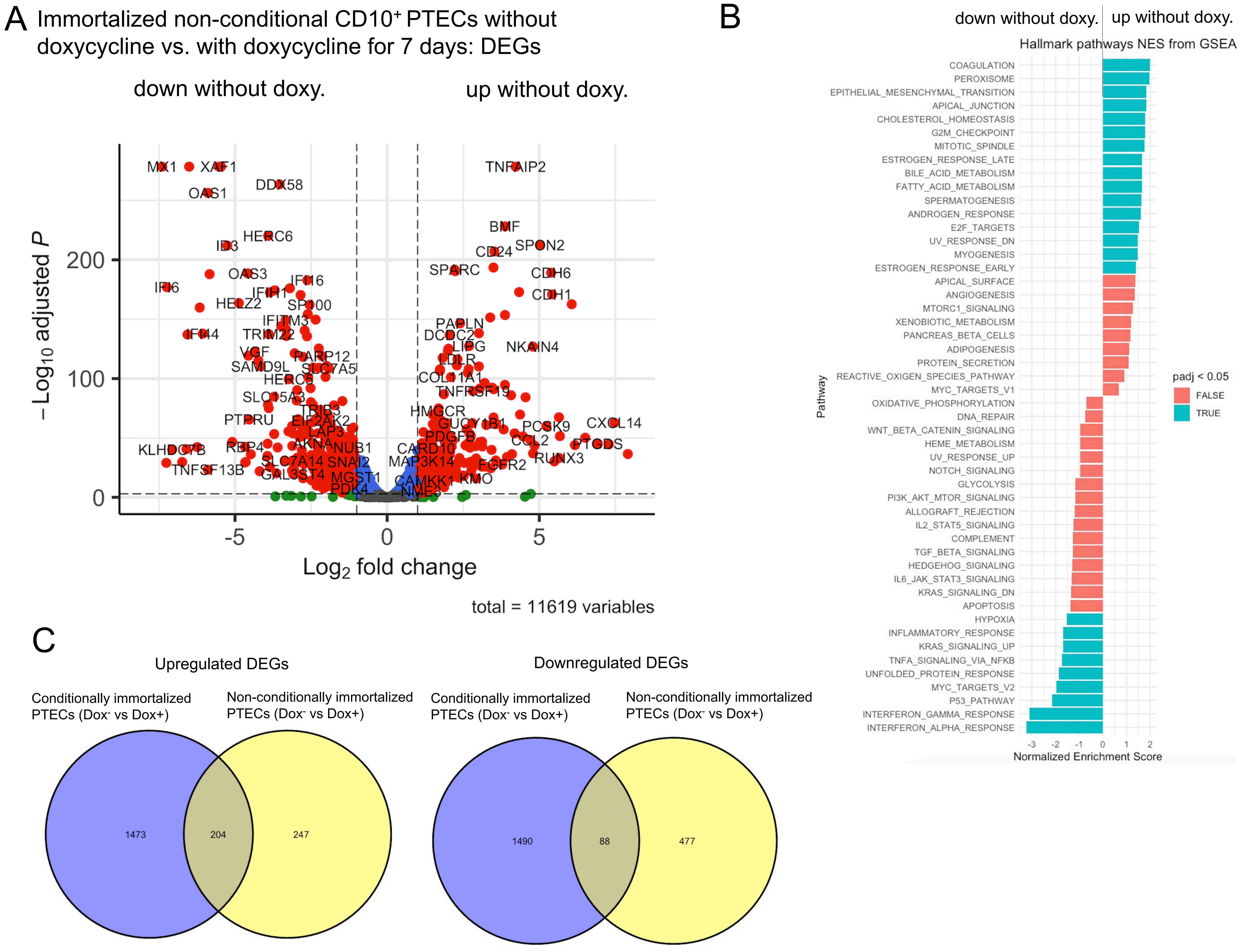

Supplemental Figure S4. Impact of doxycycline on the transcriptome of immortalized CD10+ human proximal tubule epithelial cells (PTECs). A: The volcano plot shows differentially expressed genes (DEGs) after a 7-day treatment with doxycycline. The data were obtained from bulk RNA-seq. **B:** Hallmark pathways associated with the normalized enrichment score (NES) determined via gene set enrichment analysis (GSEA). **C:** Overlap of up- and downregulated DEGs (padj < 0.05 & log2FoldChange > 1 or <-1) in both conditionally and non-conditionally immortalized PTEC lines. n=3.
